# Regulatory Role of N6-Methyladenosine (m6A) Modification in Osteoarthritis

**DOI:** 10.3389/fcell.2022.946219

**Published:** 2022-06-30

**Authors:** Ganggang Zhai, Likang Xiao, Chenyang Jiang, Songkai Yue, Meng Zhang, Jia zheng, Zeming Liu, Yonghui Dong

**Affiliations:** ^1^ Department of Orthopedics, Henan Provincial People’s Hospital, People’s Hospital of Zhengzhou University, Henan University People’s Hospital, Zhengzhou, China; ^2^ Department of Plastic and Cosmetic Surgery, Tongji Hospital, Tongji Medical College, Huazhong University of Science and Technology, Wuhan, China; ^3^ Microbiome Laboratory, Henan Provincial People’s Hospital, People’s Hospital of Zhengzhou University, Zhengzhou, China

**Keywords:** N6-methyladenosine, molecular mechanisms, osteoarthritis, biomarker, therapeutic target

## Abstract

Osteoarthritis (OA) is the most common joint disease, usually occurring in middle-aged and elderly people. However, current treatment for OA in its early stages is ineffective, and drug therapy is often ineffective in slowing the progression of the disease. In fact, a deeper understanding of the underlying molecular mechanisms of OA could help us to better develop effective therapeutic measures. N6-methyladenosine (m6A) is a methylation that occurs at the adenosine N6-position, which is the most common internal modification on eukaryotic mRNAs. The role and mechanisms of m6A in mammalian gene regulation have been extensively studied. The “Writer”, “eraser”, and “reader” proteins are key proteins involved in the dynamic regulation of m6A modifications. Recent studies on post-transcriptional regulation alone have shown that m6a modification has an important role in the development of OA. This paper summarizes the specific regulatory processes of M6A in disease and reviews the role of m6A in OA, describing its pathophysiological role and molecular mechanisms, as well as its future research trends and potential clinical applications in OA.

## Introduction

Osteoarthritis (OA) is a chronic, degenerative joint disease and is one of the most common clinical joint disorders (Stonestrom, 2018). With the increasing aging of the global population, the incidence of OA is increasing year by year. The pathology is characterized by degeneration or destruction of the articular cartilage, secondary hyperplasia of the surrounding bone, and degeneration of the synovium and meniscus. It is often characterized by pain, limitation of movement, and joint deformity (Langworthy et al., 2010). The pathogenesis of OA is still not fully understood, but numerous studies have shown that the excessive inflammatory response and apoptosis during OA progression leads to the destruction of chondrocytes, which ultimately causes the disease to progress (Chen et al., 2017). Few drugs are clinically effective in relieving OA, and there is a lack of more satisfactory treatments for patients with advanced disease other than joint replacement surgery. Therefore, great efforts are needed to further investigate the biology of osteoarthritis in order to develop new therapeutic options (Richmond et al., 2010).

Post-transcriptional modifications are important regulators of a variety of physiological processes and disease progression, and thus have received increasing attention in bioscience research (Harvey et al., 2018). Among the numerous RNA modifications, N6-methyladenosine (m6A) is the most abundant mRNA modification (Jonkhout et al., 2017). m6A RNA methylation is the addition of a methyl group to the nitrogenous base position at position six of the adenine residue of RNA (Ma et al., 2019). m6A modifications affect almost all aspects of mRNA metabolism, including maintenance of RNA stability, RNA selective splicing and translation, thereby affecting apoptosis, autophagy and immune response (Dominissini et al., 2012). With the rapid development of molecular biology and sequencing, m6A modifications have been reported to be associated with almost all cellular functions and multiple diseases. In recent years, it has been shown that m6A modification promotes the inflammatory response, chondrocyte apoptosis and extracellular matrix imbalance in OA, which ultimately leads to the exacerbation of OA. In addition to this, previous work has found that the progression of OA can be inhibited by treatment with demethylation in a mouse model (Li et al., 2021). In this paper, we briefly describe the mechanism of m6A modification and its important role in RNA synthesis, transport and translation. Next, we summarize the role of m6A modification in osteoarthritic diseases and discuss future research directions.

## N6-Methyladenosine-Related Enzymes and Their Dynamic Modification Process

**FIGURE 1:**
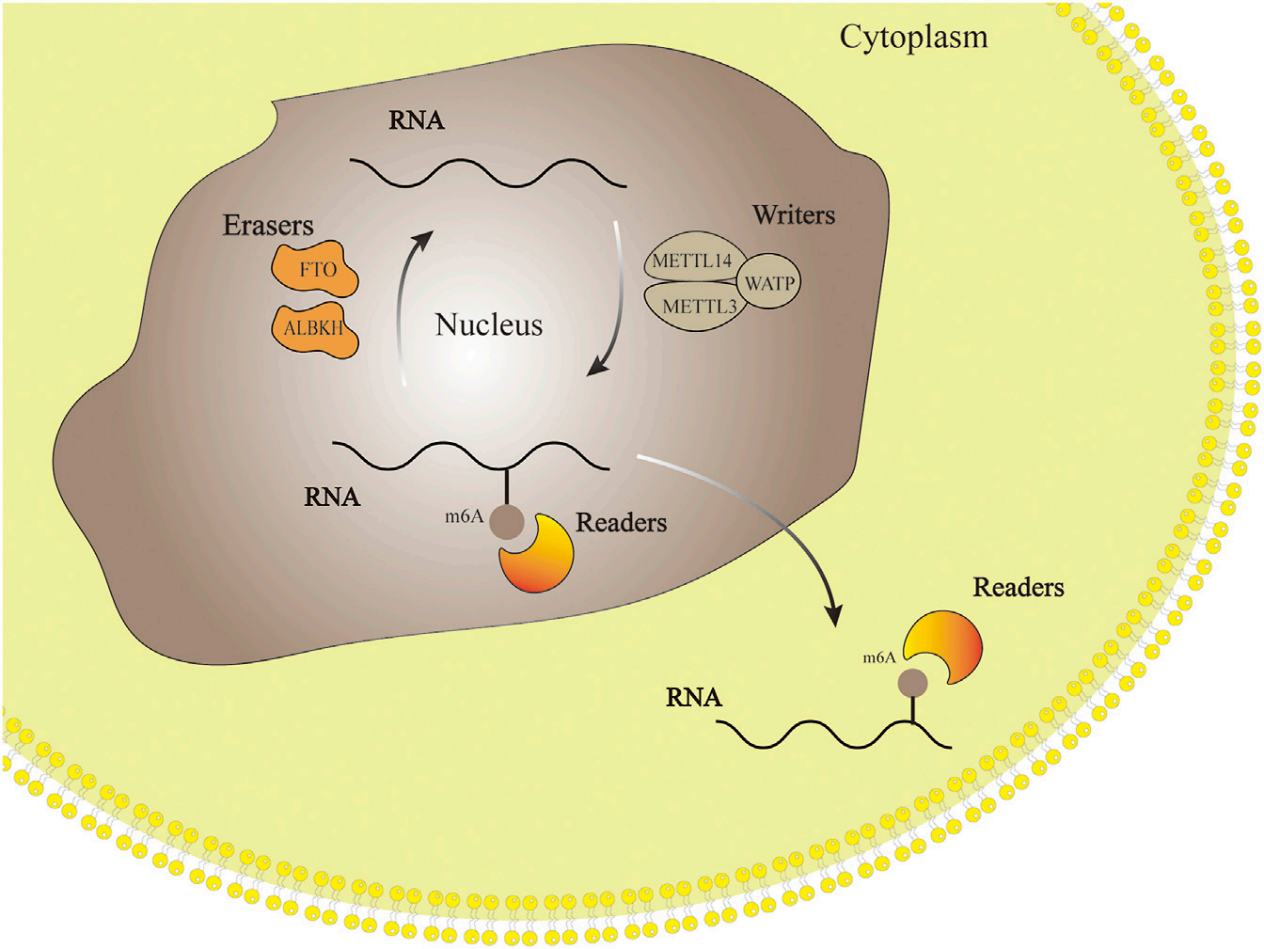
RNA methylation modification process. Writers, erasers and readers add, remove and identify methyl groups respectively. This process makes the m6A modification flexible and reversible.

M6A-related enzymes determine whether m6A is methylated or demethylated. In other words, the type of m6A-related enzymes reveals the function and significance of m6A. M6A-related enzymes include “writers”- Methylesterase, “erasers”-Demethylase and “readers”-m6A Reading Protein. The process of M6A modified RNA includes writer addition, reader recognition and eraser removal. (Wu et al., 2016). Methyltransferase like protein3 (METTL3), Methyltransferase like protein14 (METTL14), Wilms’ tumor 1-associated protein (WTAP) and other proteins can form writers with various binding proteins to regulate the methylation of RNA. On the contrary, m6A erasers protein can catalyze RNA demethylation, which is a part of m6A modification in reversible dynamic process. Readers include YTH domain family and heterogeneous nuclear ribonucleoprotein (hnRNP) family, whose role is to recognize m6A-mediated physiological behavior and influence RNA function (Zaccara et al., 2019). The dynamic process of m6A regulation is shown in Figure 1.

### N6-Methyladenosine Writers

Writer is the methyltransferase complex which is composed of the catalytic subunit METTL3 and other accessory subunits including METTL14, WTAP, VIRMA, RBM15, and ZC3H13 (Wang et al., 2016). The most important components are METTL14, WTAP, and METTL3. METTL3 can combine with methyl alone to catalyze m6A formation and promote protein translation in cytoplasm. Although METTL14 can’t catalyze the formation of m6A methyl modification independently, it can form heterodimers with METL3, and the methylation activity of this complex is higher than that of METL3 alone. Therefore, it can be seen that these proteins are not isolated in organisms, but form a complex to perform the catalytic function jointly (Liu et al., 2014). WTAP has the functions of stabilizing heterodimers, helping to locate nuclear spots, promoting RNA degradation and regulating cell differentiation and proliferation (Chen et al., 2019). RBM15 binds to the m6A complex and recruits it to specific RNA binding sites. VIRMA and ZC3H13 play a regulatory role in the process of methylation respectively (He et al., 2019).

### N6-Methyladenosine Erasers

Erasers, which are demethylases, are essential for the m6A modification to remain a dynamic and reversible process (Wang et al., 2014). Eraser can demethylate the base modified by M6A by demethylase. Two types of Erasers are known, FTO and ALKBH5. FTO is the first demethylase identified, first thought to be a gene associated with obesity, enriched in the brain, especially in neurons, and plays an important regulatory role in the central nervous system (Zhang et al., 2019). The FTO-mediated demethylation process is stepwise, m6A is first converted to N6-hydroxymethyladenosine (hm6A), then to N6-formyladenosine (f6A), and finally reduced to the original adenosine acid (Wu et al., 2015). AKKB homolog 5 (ALKBH5) is the second demethylase identified after FTO, which is highly expressed in the testis and is essential for spermatogenesis (Zhou et al., 2017). Its mediated demethylation process has no intermediate products. The differences about the m6A metabolism mechanism between FTO and ALKBH5 result in distinct biochemical outcomes and different biological functions.

### N6-Methyladenosine Readers

The main function of Readers is to recognize bases modified by m6A and regulate RNA processing, transport, translation and stability (Wang et al., 2020). Up to now, the m6A reader proteins that have been identified include the YTH family (YTHDF1, YTHDF2, YTHDF3, YTHDC1, YTHDC2), the hnRNP family (HNRNPA2B1, HNRNPC, HNRNPG) and the IGF2BP family (IGF2BP1, IGF2BP2, IGF2BP3). Among them, the YTH domain-containing protein was the first reader to be discovered (Huang et al., 2018). Human YTHDC1 has been proved to be involved in RNA splicing. In addition, it has been proved that YTHDF2 can reduce the stability of its targeted methylated mRNA transcript and control its span, while YTHDF1 can ensure the efficient expression of protein from its shared region. These studies have proved that YTHDF family proteins have potential synergistic effect in regulating gene expression (Sun et al., 2020).

Although our current understanding of the precise mechanism of m6A RNA modification is limited, various studies have examined the effects of m6A modification on the regulation of RNA transcripts.

## N6-Methyladenosine and Bone Development

**FIGURE 2:**
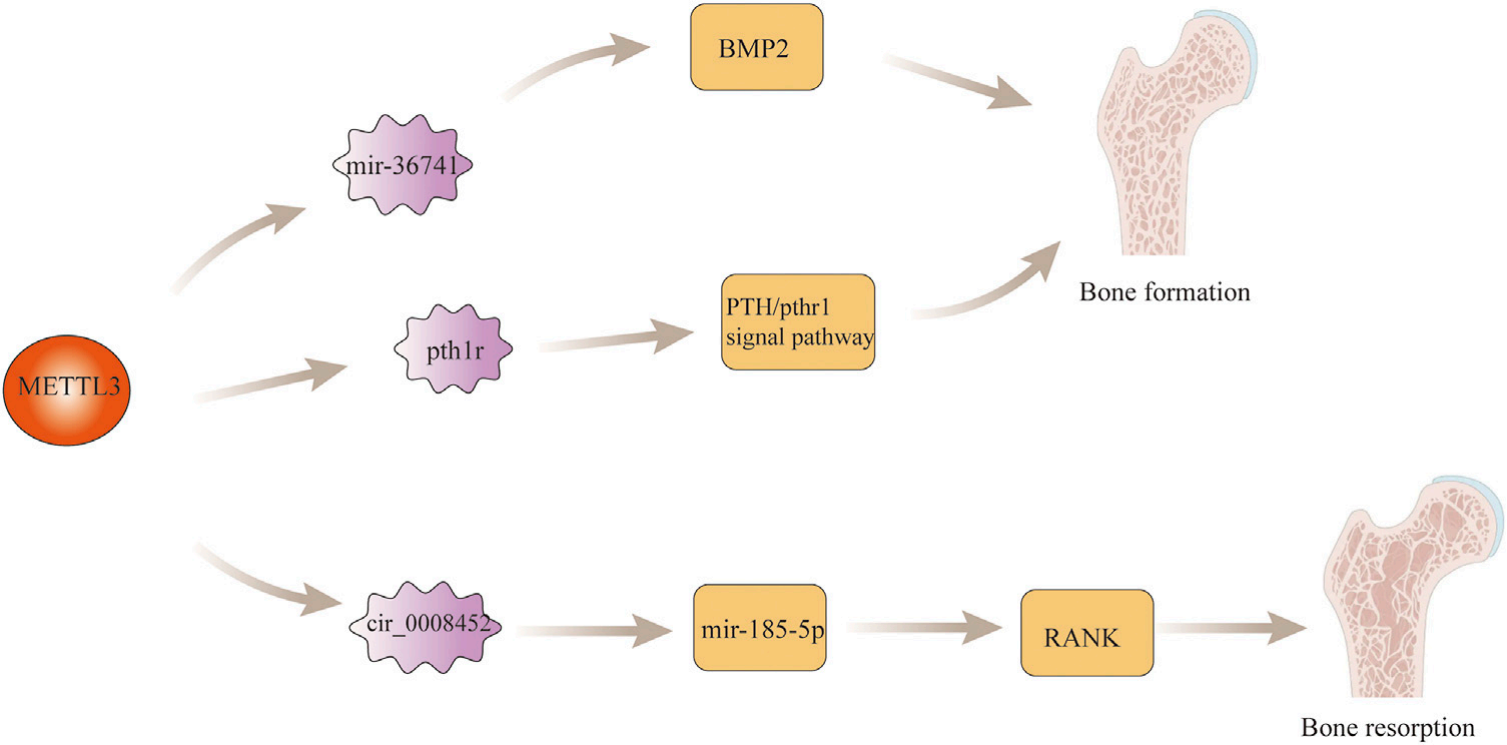
m6A modification in bone development. M6a modification regulates osteoblast differentiation by regulating MBP2 gene expression and the PTH/Pth1r signaling axis. In addition, m6a modification also promotes the process of bone resorption by regulating the expression of RANK gene.

The maintenance of normal tissue and anatomical structure of bone is the result of the complementary and balanced functions of osteoblasts and osteoclasts in bone (Nakashima, 2016). Imbalance between osteoblasts and osteoclasts often leads to lesions of bone. Found that the expression of PIWI-interacting RNA-36741 (pri-36741) and METTL3 were upregulated during the induction of osteoblast differentiation using primary human bone marrow mesenchymal stem cells (BMSCs). After human silencing the expression of Pri-36741 and METTL3, the osteoblast markers decreased, indicating that METL3 plays a regulatory role in osteoblast differentiation. Ultimately, it was found that in terms of specific regulatory mechanisms, the pri-36741-PIWIL4 complex binds directly to METTL3, inhibits METTL3 methylation regulation of bone morphogenetic protein-2 (BMP2), and promotes BMP2 mRNA and protein expression, thereby promoting osteoblast differentiation Liu et al. (2021). In addition to this, found that the level of m6A in bone marrow mesenchymal stem cells (BMSCs) was decreased in paraffin sections of femurs from mice knocked out of Mettl3, and found that the trabeculae of the distal femoral epiphysis of mice exhibited a pathological phenotype similar to that of osteoporosis by μCT. After the study, it was found that knockdown Mettl3 mice BMSCs had poor osteogenic differentiation ability, which led to a decrease in osteoblasts and osteocytes, bone loss, and even the development of osteoporosis. In terms of specific regulatory mechanisms, mettl3-deficient BMSCs are responsible for osteoporosis through the regulation of the PTH/Pth1r signaling axis to inhibit osteoblast differentiation Wu et al. (2018). In recently, Wang et al. found that RNA methylase METTL3 acted on the 1956bp m6A functional site in circ_0008542, which promoted the competitive binding between circ_0008542 and miRNA-185-5p, resulting in the increase of target gene RANK and the initiation of osteoclast bone resorption (Wang et al., 2021). This indicates that m6A is fully involved in the regulation of bone homeostasis. If bone homeostasis is destroyed, many bone metabolic diseases such as osteosarcoma, osteoporosis and osteoarthritis may occur. The specific role of m6A modification regulatory mechanism in bone development is shown in Figure 2.

### Dysregulation of N6-Methyladenosine Writers in Osteoarthritis

Many findings have shown that mettle3 is aberrantly expressed in cartilage in OA patients or OA mouse models. Simulated *in vitro* inflammatory state using IL-6-treated chondroprogenitor cells ATDC5 and found that METTL3 mRNA levels, percentage of m6A methylation in total mRNA and apoptosis rate of chondrocytes were increased compared to the chondrocyte group not given IL-6 treatment. Then silencing METTL3 expression revealed reduced chondrocyte apoptosis, suppressed inflammatory response, suppressed cellular inflammatory response signal NF-kB and reduced extracellular matrix (ECM) synthesis. Furthermore, in an *in vivo* experiment in a mouse model of OA, injection of methylation inhibitors affected METTL3 mRNA expression but significantly inhibited total m6A levels, inflammatory conditions, and ECM degradation Liu et al. (2019). Additionally, Chen et al. found that cellular senescence markers and METTL3 mRNA expression were upregulated and autophagy markers were downregulated in synovial tissues from OA patients and *in vitro* OA models. And *in vitro* cartilage experiments revealed that senescent synovial tissues were able to cause cartilage dysfunction and aging. To assess whether autophagy mediates FLS senescence, it was finally found that metttl3-mediated ATG7 m6A modification could inhibit cellular autophagy, and the next inhibition of autophagy was through the senescence regulator (GATA4) to regulate the process of cellular senescence, thus affecting the progression of OA (Chen et al., 2022a). However, found that METTL3 expression was reduced in knee cartilage of OA patients compared to cartilage of femoral neck fracture patients, and this was also demonstrated in an OA model constructed using IL-1β-treated SW1353 cells. Next, it was found that overexpression of METTL3 increased the level of inflammatory factors, promoted the expression of p-65 protein and p-ERK, activated the NF-κB signaling pathway and thus aggravated OA progression, and overexpression of METTL3 also regulated the balance between TIMPs and MMPs, thus affecting the degradation of ECM. The contradictory results may result from the limited potential of SW1353 cells as a cell line to mimic primary human chondrocytes and the differences in femoral cartilage compared to knee cartilage Sang et al. (2021).

### Dysregulation of N6-Methyladenosine Erasers in Osteoarthritis

The FTO gene, encoding a 2-oxoglutarate-dependent nucleic acid demethylase (Gerken et al., 2007), has been demonstrated in related studies to be an obesity-associated gene. Some studies have suggested that the FTO gene mediates the progression of OA through obesity. However, some argue that the causal relationship between obesity and OA is difficult to establish (Pang et al., 2013), thus rejecting this theory. For example, compared patients with temporomandibular joint osteoarthritis (TMJOA)with the general population and concluded that the frequency of the FTO polymorphism allele was significantly different between the two groups, while the differences in weight, age, and sex were not statistically significant Takaoka et al. (2022). Yang et al. found that lncRNA AC008 was highly expressed in human OA cartilage samples, and found that aberrant expression of AC008 affected OA progression by regulating chondrocyte viability, chondrocyte apoptosis and ECM degradation. To investigate the specific regulatory mechanism of AC008, miR-328-3p was suggested to be the target of AC008 by bioinformatics analysis and dual luciferase reporter gene assay; while AQP1 and ANKH were the targets of miR-328-3p. In addition, the study verified the reversal of chondrocyte apoptosis by decreasing m6A levels of AC008 after FTO overexpression in chondrocytes, and finally concluded that FTO-dependent m6A demethylation-mediated upregulation of AC008 promotes osteoarthritis progression through the miR-328-3p-AQP1/ANKH axis (Yang et al., 2021).

### Dysregulation of N6-Methyladenosine Readers in Osteoarthritis

**FIGURE 3:**
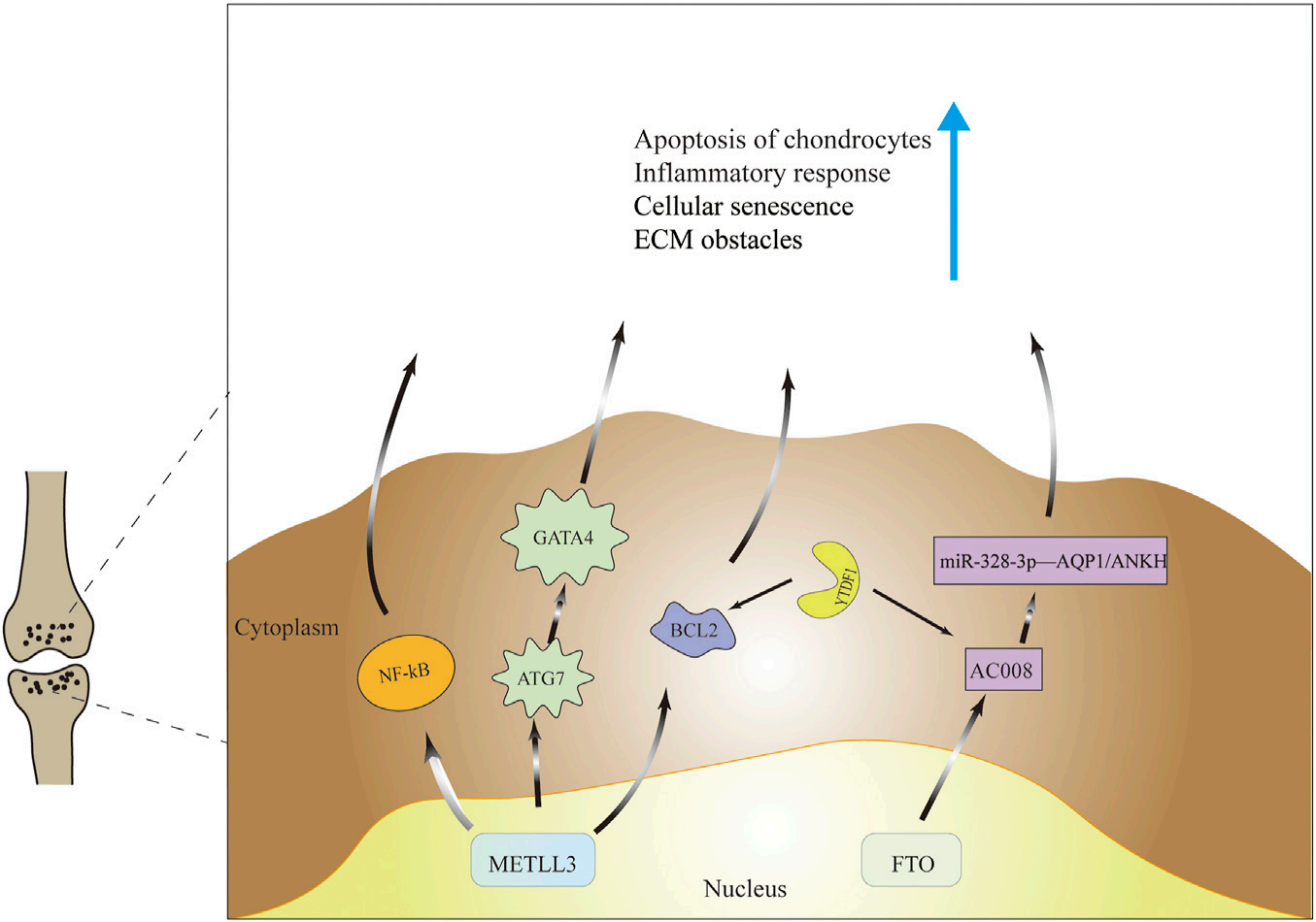
Molecular mechanisms of m6a modifications in the pathogenesis of OA. M6A modification ultimately cause cartilage apoptosis, cellular senescence, increased inflammatory response and extracellular matrix imbalance through regulation of gene expression and signaling pathways.

The main mechanism by which M6A acts is through the recruitment of m6A binding proteins (Meyer and Jaffrey, 2017). In fact, studies on m6A readers in OA are currently lacking. Recently, in a mouse cartilage OA model, demonstrated that Mettl3 was highly expressed in cartilage and caused apoptosis and autophagy in chondrocytes by directly interacting with Bcl2 mRNA and increasing Bcl2 mRNA methylation levels. It was also confirmed by protein-RNA interaction predictor (PRIdictor) analysis and mRNA stability test that Bcl2 mRNA methylation was mediated by YTHDF1 to produce a regulatory effect on OA He et al. (2022). As to whether the hnRNP family is related to the molecular mechanism of OA, no relevant studies have been found and further exploration is worthwhile. The regulatory mechanism of m6A is shown in Table 1 and Figure 3.

**TABLE 1:** Roles of m6A regulators in osteoarthritis.

m6A Regulator	Regulation	Mechanism	References
METTL3	UP	METTL3 accelerates OA by affecting the cellular inflammatory response signal NF-kB and the degradation of the extracellular matrix (ECM)	Liu et al. (2019)
METTL3	UP	Metttl3-mediated ATG7 m6A modification regulates autophagy- GATA4 axis OA progression	Chen et al. (2021)
METTL3	DOWN	METTL3 may be involved in the progression of osteoarthritis by affecting ECM degradation and modulating the inflammatory response	Sang et al. (2021)
FTO	UP	The FTO allele is a risk factor for temporomandibular joint osteoarthritis (TMJOA), which does not mediate OA progression through obesity	Takaoka et al. (2021)
FTO	UP	FTO-dependent m6A demethylation-mediated AC008 upregulation promotes osteoarthritis progression via miR-328-3p-AQP1/ANKH axis	Yang et al. (2021)
YTHDF1	UP	Mettl3 mediates Bcl2 stabilization through ythdf1-mediated m6A modification, thereby inhibiting chondrocyte apoptosis and autophagy during inflammation	He et al. (2022)

### N6-Methyladenosine and Other Bone Disease

As the most common RNA modification in mammals, m6A modifications are also common in other skeletal system diseases. In spinal degenerative diseases, many studies have suggested a regulatory role for m6A modifications. Demonstrated that ALKBH5-mediated mRNA demethylation accelerated intervertebral disc (IVD) aging through *ex vivo* experiments. AlKBH5 caused disc aging and nucleus pulposus degeneration by demethylating DNMT3B transcripts Li et al. (2022). Found that in the nucleus pulposus of patients with disc degeneration, the mRNA content of MAT2A was lower than normal, while the mRNA of the demethylase metttl16 was higher. Following this, *in vivo* and *in vitro* experiments were constructed to conclusively demonstrate that m6A modification of MAT2A pre-mRNA by METTL16 may cause increased apoptosis in the nucleus pulposus and intervertebral disc degeneration under oxidative stress Chen et al. (2022b). In addition, many studies have confirmed that m6A modification has an important regulatory role in osteosarcoma (Patil et al., 2016). Identified METTL3 as an oncogene in osteosarcoma. Knockdown of METTL3 inhibited the proliferation, migration and invasion of human osteosarcoma cell lines SAOS-2 and MG63 by suppressing m6A methylation levels and the expression of ATPase family AAA structural domain protein 2 (ATAD2) Zhou et al. (2020). Recently, Yuan et al. showed that the demethylase ALKBH5 can silence the pre-miR-181b-1/YAP signaling axis, thereby inhibiting tumor progression in osteosarcoma. In addition, upregulation of ALKBH5 expression also contributes to chemoresistance in osteosarcoma patients and predicts worsening of their metastasis-free survival (WANG et al., 2019).

## Discussion

### Clinical Value of N6-Methyladenosine for Osteoarthritis

Given that abnormal m6A modifications are closely related to cartilage degeneration, cartilage apoptosis, and inflammatory response in the pathogenesis of OA (Chen et al., 2021). Therefore, m6A modification may bring new breakthroughs for the early diagnosis as well as the treatment of OA. On the one hand, many studies have pointed to the presence of m6A hypermethylation levels and dysregulation of m6A-related enzymes in cartilage or synovial samples from OA patients (Zhang et al., 2020). However, because cartilage and synovial samples are difficult to obtain, there is a lack of studies to confirm the presence of abnormal expression of m6A modification-related markers in the peripheral blood of OA patients, so exploring whether such differences exist could be the next research focus, which is important for the early diagnosis or screening of OA. On the other hand, the current treatment modalities for OA in the world are still mainly conservative and surgical; conservative treatment includes lifestyle changes and medication, and surgical treatment includes arthroscopic debridement and joint replacement surgery (Katz et al., 2021). But the current mainstream medications have no significant effect on stopping the progression of OA, while surgical treatment is more applicable to patients with advanced OA who have lost joint function. Therefore, it is necessary to develop new therapeutic strategies for OA. In a mouse model of OA, m6A-related inhibitors have been shown to be effective in improving the inflammatory status and degradation of ECM and cellular aging in OA, thereby slowing down the progression of the disease. Unfortunately, however, the development of m6A-related drugs is only at the experimental stage, and relevant studies on clinical patients are lacking. Further studies are needed to investigate whether m6A inhibitors can effectively inhibit the progression of OA patients.

### Conclusion and Future Perspective

M6A modification has emerged as an important factor in the development and progression of OA. It can accelerate or inhibit OA progression in different ways. There is no doubt that the emergence of m6A regulation provides new insights into the molecular mechanisms of OA and may contribute to the development of new and more effective therapeutic approaches. However, the full understanding of the mechanisms of m6A modifications in OA is still in its infancy, and several knowledge gaps remain. Firstly, the role of m6A-related regulatory factors in OA, other than METTL3, has been less studied. Secondly researchers have noted the potential of m6A as a therapeutic target for OA, but few studies have focused on the clinical application of effective and specific drugs targeting the m6A enzyme. All, it is necessary to accelerate its translation to the clinic.
